# The Role of Music Therapy for Children Undergoing Cancer Treatment in Singapore

**DOI:** 10.3390/healthcare9121761

**Published:** 2021-12-20

**Authors:** Kayla Carissa Wong, Beron W. Z. Tan, Jasper W. K. Tong, Mei Yoke Chan

**Affiliations:** 1 Child Life, Art and Music Therapy Programmes (CHAMPs), Rehabilitation Centre, KK Women’s and Children’s Hospital, Singapore 229899, Singapore; 2 Psychology Service, KK Women’s and Children’s Hospital, Singapore 229899, Singapore; beron.tan.w.z@kkh.com.sg; 3 Allied Health Office, KK Women’s and Children’s Hospital, Singapore 229899, Singapore; jasper.tong.w.k@singhealth.com.sg; 4 Haematology/Oncology Service, KK Women’s and Children’s Hospital, Singapore 229899, Singapore; chan.mei.yoke@singhealth.com.sg

**Keywords:** music therapy, childhood cancer, pediatric oncology, rehabilitation, psychosocial, functional

## Abstract

The aim of this study was to explore the benefits of music therapy (MT) for children with cancer over the course of their treatment in an acute paediatric hospital setting in Singapore. Twenty-five children undergoing cancer treatment received MT sessions as part of a multidisciplinary team rehabilitation intervention from March 2017 to January 2020. A total of 37 individualised goals were developed by the music therapist for the cohort. Goals were scored via the Goal Attainment Scale at 3-month intervals up to 1 year. Descriptive statistics and correlation analysis were used to evaluate the findings. The rate of goal achievement was 89.2% over 180 MT sessions (*M* = 7.20, *SD* = 6.45). Children diagnosed with brain tumours had the highest frequency of MT sessions (*M* = 9.11, *SD* = 7.79). Most of the goals targeted the regulation of mood and morale through music. There was a positive correlation found between goals and sessions (*r*_s_ = 0.56, *p =* 0.004). Age of the children was not correlated with the number of sessions received (*r*_s_ *=* −0.19, *p =* 0.354). MT has been found to be an accessible and effective intervention in addressing functional and emotional goals for children across all ages who are undergoing cancer treatment.

## 1. Introduction

The World Health Organization reported an approximation of 400,000 children being diagnosed with cancer every year [1]. The most prevalent types of childhood cancer comprised leukemias, brain cancers, lymphomas and solid tumours [1]. Music therapy (MT) has been an integral part of childhood cancer care since 1973 [2,3]. However, literature on music therapy for childhood cancer care has remained scarce, with more information provided for music therapy in cancer care for adults [4].

MT is usually indicated as a complement to or integrated with conventional medical therapy in cancer care [5]. Non-pharmacological treatments for managing symptoms are preferred for children undergoing cancer treatment due to its low risk of side effects. MT is one such low-risk intervention that has increasingly been indicated in psycho-oncological support for its benefits in addressing symptoms such as anxiety, low mood, and pain [6]. The Association of Music Therapy (Singapore) defines the profession as follows: “Music therapy is the scientific use of music interventions within a therapeutic relationship towards observable or measurable functional, educational, rehabilitative or well-being outcomes by a credentialed professional [7].”

Distressing side effects from cancer treatment include fatigue and pain, weakness, loss of hair, nausea and vomiting, loss of appetite, mouth ulcers, skin rashes, and infections [8]. Common stressors faced by children undergoing cancer treatment are related to being in an unfamiliar hospital environment full of strangers and being separated from their siblings, family members and friends for prolonged periods [8,9]. Multiple hospitalizations for treatment over a long period of time is also disruptive for children’s typical development, activities and social life which can impact their quality of life [10]. These prolonged periods of isolation can lead to depression and anxiety [11]. Some other ways that children may be psychologically affected are worrying about the future, their appearances, and feeling different from their peers [10]. Survivors of childhood cancer have also been found to be at risk of psychological late effects and neurocognitive deficits i.e., attention, verbal and working memory, and processing speed [12]. 

It was reported that about one-quarter of children undergoing cancer treatment will struggle with psychological disorders such as major depressive disorder, anxiety disorders, and/or post-traumatic stress disorder [10]. These disorders in turn can also negatively influence their symptom management and compliance with treatment [10]. Interestingly, survivors of childhood cancer showed higher levels of anxious symptoms even if they were reported to be doing well psychologically during cancer treatment [12]. Furthermore, it has also been noted that while survivors of childhood cancer do not typically complain of psychological symptoms, they have been reported to have higher levels of internalizing emotional symptoms and social withdrawal [12].

The Standards of Care for Children with Cancer developed by the European Society for Pediatric Oncology recommended that every child with cancer should have an option for psychological support throughout their cancer journey [13]. Studies have encouraged interventions that can provide these children the opportunity for choice and freedom, exploration of new areas of expertise or mastery, and encouragement to engage in activities that can provide normalization; all of which MT has the diversity to address [14].

Music has the potential to activate emotions, images, memories, and associations, bypassing psychological blocks a person might have regarding an issue [15]. Imagery evoked by music can offer an emotional safe space and respite from the harsh effects of cancer and its treatment [16]. The aesthetic elements of music can also offer comfort and peace when patients are distressed [16]. Music interventions can provide the opportunity for making independent choices, connections, and intrinsically evoke physical, emotional, and social expression [2,9,11]. MT involves music listening and/or music-making experiences within a therapeutic relationship between the music therapist and patient [2]. Music therapists are trained to clinically assess a child’s prior traumas, medical history, and existing wellness and coping strategies to design and implement a personalized treatment plan to support the child through their cancer journey [3].

This study explored the benefits of MT for children with cancer and described the profile and common goals of children who required MT while undergoing cancer treatment. MT intervention was delivered as part of the rehabilitation program to support children with cancer in improving functional outcomes. We aim to examine the (a) profile of patients who were referred for MT (b) frequency of accessing MT over a child’s cancer treatment (c) goals and objectives of MT services for children undergoing cancer treatment and (d) efficacy of MT based on goals achieved.

## 2. Materials and Methods

### 2.1. Participants

**Figure 1:**
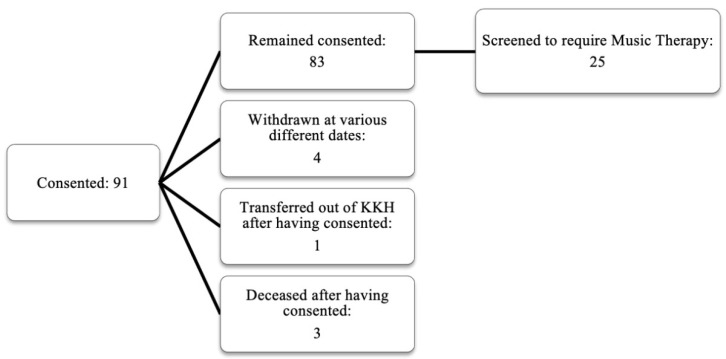
Participants screened and eligible for music therapy under the PSCP.

Ninety-one children were recruited as part of the Psychosocial and Supportive Care Programme (PSCP), which includes psychology, dietetics and nutrition, and rehabilitation (comprising physiotherapy, occupational therapy, speech and language therapy, and MT) services to support all children undergoing cancer therapy at KK Women’s and Children’s Hospital, Singapore’s largest tertiary hospital catered to children. This is a single cohort observational study that included children between the ages of 2–17 years who were newly diagnosed with a malignant solid tumour, blood cancer, or brain tumour from March 2017 to January 2020; Singaporean and/or permanent residents; and were receiving treatment in the hospital. Exclusion criteria included non-Singaporeans or non-permanent residents, children aged less than 2 years and more than 17 years, and children with relapse cancer. All children were subsequently screened to ascertain whether they required MT. Twenty-five children (age; *M* = 8.36 yrs., *SD* = 4.66 yrs.) received MT from March 2017 to January 2020. These children constituted 30.1% (Figure 1) of the total PSCP participants. 

### 2.2. Screening Procedure

Eligible children for the PSCP study were recruited at the stage of diagnosis and consent was taken during the family conference where the individualized medical treatment plan was discussed. To ensure the children were provided with early rehabilitation to meet their needs, newly admitted children with cancer were screened daily by a therapist such as the physiotherapist, occupational therapist, or speech and language therapist. The protocol involved the therapist approaching the child and their parent/main caregiver to introduce the rehabilitation services provided. The attending therapist screened and ascertained if children were suitable for MT via any one of these two main questions: (1) Child is moody/withdrawn? If yes, refer to MT; (2) Child is unresponsive/non-compliant to treatment and therapy. If yes, refer to MT. The attending therapist would then have a discussion with the child and their parent/main caregiver regarding how the child could benefit from MT (i.e., optimization of rehabilitation goals through increased motivation and/or comfort through music). Once the child and their parent/main caregiver agree to a referral, the music therapist would then meet the child for MT.

The music therapist would begin by building rapport with the child and getting to know his or her musical preferences and background. Based on the individual child’s preferences, ability and age appropriateness, the music therapist would then offer a suitable MT intervention(s). MT interventions as defined by Bruscia [17] include receptive (active music listening, song choices, lyric discussion), recreative (vocal or instrumental music making of precomposed music), improvisational (spontaneous vocal or instrumental music making), and compositional (song compositions based on client’s choices of lyrics and chords supported by the music therapist) [18]. These interventions can be used reflexively by the music therapist as an integrative experience based on the context and need of the child [18]. The music therapist would also provide an emotional space for the child to verbally process thoughts and emotions evoked by the music [16]. 

The children were engaged about one to three times a week by the attending music therapist based on their clinical needs. Frequency of sessions were dependent on the treatment schedule and agreed upon by the music therapist and the child and family. Tools included in MT were guitar and/or keyboard, percussion instruments (i.e., shakers, drums, etc.), and technology that allowed the playing or making of recorded music (i.e., tablet, phone, laptop).

### 2.3. Study Design

This paper detailed an observational study that used frequency analysis and correlation to quantitatively explore the benefits of MT services for children undergoing cancer treatment in an acute paediatric hospital setting in Singapore. 

### 2.4. Measurements

The Goal Attainment Scale (GAS) is an approach to measure the process of achieving established unique individualized goals for patients following an intervention [19]. This scale was chosen as it can be used easily and quickly while considering the goals of the child and family. GAS was developed by Thomas Kiresuk and Robert Sherman in 1968 for the mental health population and has since been widely used in areas such as counselling and family therapy, substance abuse treatment, rehabilitation, and MT [19]. GAS is suitable to be applied to any MT approach as it is not based on any specific theoretical orientation, population, treatment type or assessment tool [19].

The process of rating the GAS in MT was similar to those reported in Bovend’Eerdt, Botell and Wade [20], and Carpente [19]. First, individual goals were developed by the music therapist according to the child’s needs based on the SMART framework (specific, measurable, attainable, relevant, and time-bound). Second, the individualized goals were rated on a 5-level rating scale ranging from −2 to +2. Specifically, −1 represents the goal at baseline and 0 indicates that the goal has been adequately met; while +1 and +2 represent that the goal has been exceeded, and −2 represents a regression in patient’s baseline (Table 1). 

The determination of the GAS outcome level at the point of evaluation is reliant on the therapist’s clinical experience, perception, and rational discernment of the patient [19]. Lastly, the goals were assigned to one of the three domains as listed in the World Health Organization by the International Classification of Functioning (WHO-ICF), Disability and Health [21]. The WHO-ICF domains consisted of (1) Body Functions/ Impairments, (2) Activity and Participation/Activity Limitations and Participation Restrictions, (3) Environmental Factors. Goals were written for children during the period that they were assessed by the music therapist to require MT services. A total of 37 goals were written from March 2017 to January 2020. Goals written were found to only fall under two WHO-ICF categories; “Body Functions/Impairments” and “Activity and Participation/Activity Limitations and Participation Restrictions.”

### 2.5. Data Collection

Each child had SMART goals written by the music therapist within the GAS at the point of clinical assessment. GAS was scored every 3 months up to 1 year through the child’s treatment process, where applicable. Goals that received a 0, +1, +2 at the point of evaluation (end of each 3-month interval) were considered achieved while goals that received a −1 or −2 at the point of evaluation were considered not achieved. 

### 2.6. Analysis

This study used frequency analysis which included cross tabulation, and Spearman’s rank-order correlation to explore the associations between age, goals, and sessions. The level of statistical significance was set at *p* < 0.05. 

## 3. Results

Demographics of the children who received MT including diagnosis, gender, age, and race were collected (Table 2). Over the course of the study, the children received a total number of 180 sessions. The highest number of sessions was received by children with brain tumours, followed by blood cancer, and finally solid tumours (Table 3). It was observed that 15 children had a single goal, followed by nine children who had two goals, and one child who received four goals. There was a significant positive correlation between goals and sessions (*r*_s_ = 0.56, *p =* 0.004). There was no correlation between the age of the children and the number of sessions received by the children (*r*_s_ = −0.19, *p* = 0.354). 

With GAS being scored every 3 months, the rate of goal achievement was observed across time. Overall, 33 out of 37 goals (89.2%) were achieved. The written goals were mapped across two WHO-ICF components, namely, “Body Functions/Impairments”, and “Activity and Participating/Activity Limitations and Participation Restrictions”. The former WHO-ICF component had three subdomains, while the latter WHO-ICF component had five subdomains. No goals were found on the “Environmental Factors” domain. The details of these subdomains and examples of the written goals were displayed in Table 4. 

### 3.1. WHO-ICF Component 1: Body Functions/Impairments 

Within the WHO-ICF component 1, there were 18 written goals. The highest number of goals achieved was under the subdomain of mental functions, which included emotional functioning. Specifically, 16 out of 18 goals (88.9%) that were achieved under this subdomain were on the regulation of children’s mood and morale through music. The remaining two goals (11.1%) achieved were under the subdomains of voice and speech functions, and sensory functions.

### 3.2. WHO-ICF Component 2: Activity and Participating/Activity Limitations and Participation Restrictions

Within the WHO-ICF component 2, there were 19 written goals; 16 out of these 19 goals (84.2%) were achieved. Goals addressing “interpersonal relationships (informal and formal relationships)” such as enhancing the interactions between children and nursing staff or other therapists through music, five goals targeting “general tasks and demands (daily routines, handling stress, managing behaviour),” three goals covering “major life areas (education, work, engagement in play),” two goals addressing “community, social, and civic life (community/social activities, recreation/leisure)” and two goals focusing on “communication (receptive, productive, using communication devices).” Three out of four of the goals that were not achieved were in the subdomain of “interpersonal relationships (informal and formal relationships)”, which is under the domain of “Activity and Participation/Activity Limitations and Participation Restrictions.” 

## 4. Discussion

Children with cancer experience varying problems which may be overlooked, especially on their emotional functioning [8]. Our study found that 30.1% (N = 25) of participants received MT with most goals targeting the regulation of the children’s mood and morale through music. This finding is comparable to Sargin Yildrim et al. [8] who noted that 36% of children with cancer undergoing treatment were detected to have mood symptoms. 

Studies with children undergoing high-dose chemotherapy with autologous stem cell transplantation have discovered that those who received MT experienced significantly fewer disturbances in mood, improvement of pain perception scores, and an overall positive effect on depression and anxiety [6]. Music experiences have been shown to exhibit strong ties with reward and learning, attention, memory, and emotions which in turn allows the music therapist to naturally cater to psychosocial needs [6,22]. Emotionally significant events are embedded into memory, and studies show that music can enhance the expression of events with greater meaning and emotion than speaking [23]. The activation of dopaminergic neurons through music experiences can also regulate and increase arousal, as well as motivation, demonstrating how music can be used as a coping tool to help in relieving feelings of depression and anxiety [22,23]. Endorphins are other neurotransmitters that have been scientifically reported to be released through music experiences, effectively masking pain and potentially positively influencing other related symptoms [4].

Our study found that children with brain tumours had the highest frequency of MT sessions compared to those with blood cancer or solid tumour. This finding may suggest that children with brain tumours may require more support offered by MT. Indeed, studies had reported that children diagnosed with central nervous system tumors had lower quality of life scores as compared to those who were diagnosed with lymphoma and other cancers (e.g., [8]). In our study, the wide spectrum of goals addressed by MT demonstrated its therapeutic versatility for approaching health in a holistic manner. Such versatility was also reflected in the literature with several MT studies reported to have made a significant impact in a wide range of areas such as anxiety and depression, mood and affective state, nausea, fear, physiological responses, and pain [18]. 

Although most of the goals in our study were achieved (i.e., 89.2%), there were no clear patterns amongst diagnosis, number of goals and sessions, age, race, and gender observed which can explain why 10.8% of the goals were unable to be achieved. However, it is noteworthy that three out of four of the goals that were not achieved were in the subdomain of “interpersonal relationships (informal and formal relationships)”, which is under the domain of “Activity and Participation/Activity Limitations and Participation Restrictions.” Such findings suggest that more psychosocial support may be required to fulfil the complex needs of children with cancer (e.g., family therapy). 

Considering the multitude of challenges a child undergoing cancer treatment experiences [8,9,10,11], it is important for children to have interventions that can adequately address emotional needs. Music therapy meets this requirement in offering effective intervention as our study has shown that the majority of children were able to experience meaningful achievements of bodily functions and activity participation. Both components are essential elements in maintaining the holistic wellbeing of an individual child [8].

### Study Limitations

Other limitations for this study included a limited sample size which made it difficult to compare differences among diagnosis, age groups, gender, and other variables. Moreover, although our study found that children with brain tumors had the highest frequency of MT sessions relative to blood and solid cancers, further investigation with a larger sample size is needed to validate this finding. 

The Singapore healthcare financing system operates on a subsidized fee-for-service model while this current study is part of a fully funded psychosocial program. Hence, the accessibility and acceptance of MT sessions may differ if the family of the children had to bear the cost of therapy, limiting the generalizability of session frequency and acceptance of MT by the children and their families. Within the constraints of the funding provision, MT was only accessible to children undergoing cancer treatment in the inpatient setting. Thus, children who are on a treatment protocol that do not require hospitalizations would not have received MT (e.g., day therapy outpatient appointments). Future research efforts may also consider exploring how children and their families in Singapore receiving MT as part of cancer care perceive the service to be meaningful to them (e.g., via questionnaires). 

Additionally, no pre- or post-efficacy measures were used in this study. Goals were written and assessed by the music therapist, with minimal input from the children and family. Hence, the type of goals written may reflect the music therapist’s preference in terms of approach and practice, possibly differing if there was another music therapist involved, or participant-reported goals. Albeit speculative, this limitation may also influence the absence of the third WHO-ICF component (i.e., Environmental Factors) in this study, which referred to physical, social, and attitudinal factors that may serve as a barrier or enabler to a person’s functioning [21]. Future studies can improve on structured methods in writing goals and ascertain goal attainment through structured assessment involving participants and their families. In addition, future studies may consider exploring the influence of music therapist’s approach and/or participant’s input on the type of therapy goals and outcomes, as well as consider observing the specific MT interventions and types of music used for the various age groups, needs and diagnoses. Given that MT has been found to play a major part in supporting children’s mood and morale through treatment in this study, future studies may also consider evaluating depression scores pre and post interventions. Notwithstanding the limitations, this paper is the first known MT study on children with cancer in Singapore and our main finding that 89.2% of the children with cancer achieved their therapeutic goals within the set period is encouraging. 

## 5. Conclusions

Music therapy is an accessible and effective intervention that has therapeutic versatility in supporting functional and emotional goals for children of different ages who are undergoing cancer treatment. MT in paediatric cancer care involves a therapeutic relationship between the music therapist and child, where music is used to address the child’s various needs. Despite the study limitations, this paper adds to the literature in determining the role of MT services for children undergoing cancer treatment in Singapore. The most common need addressed by MT was found to be the regulation of the child’s mood and morale. Additional MT sessions may be required, especially for children with brain tumours, although this finding would need to be further investigated. 

Overall, this study has demonstrated that MT has the potential in addressing functional and psychosocial challenges for children undergoing cancer treatment and should be considered as an integral part of holistic paediatric cancer care.

## Figures and Tables

**Table 1:** Goal Attainment Scale (GAS) scoring system (Carpente, 2018).

Score	Description
+2	Goal achieved most favorably
+1	Goal achieved more than expected
0	Goal achieved
−1	Baseline
−2	Regression of baseline

**Table 2:** Demographics of participants who received music therapy services.

Demographic	Value	Frequency	%
Diagnosis	Solid Tumour	7	28
	Blood Cancer	9	36
	Brain Tumour	9	36
Gender	Male	18	72
	Female	7	28
Age	2–6 years old (Preschool age)	11	44
	7–12 years old (Primary school age)	8	32
	13–17 years old (Secondary school age)	6	24
Race	Chinese	15	60
	Malay	7	28
	Indian	1	4
	Others	2	8

**Table 3:** Frequency of music therapy sessions.

Cancer Type	No. of Participants	Mean (SD)
Overall	25	7.20 ± 6.45
Solid Tumour	7	3.28 ± 2.60
Blood Cancer	9	8.33 ± 5.73
Brain Tumour	9	9.11 ± 7.79

**Table 4:** Examples of GAS goals within the different World Health Organization by the International Classification of Functioning (WHO-ICF) domains from March 2017 to January 2020.

Domain	Subdomain	Example of SMART Goal
Body functions/Impairments	Mental functions (temperament, energy, attention, memory, emotion, cognition)	For child to engage in musical engagement for 20 min for maintenance of mood and morale through treatment
	Sensory functions and pain (seeing, hearing, taste, smell, touch, pain)	For child to shift perception of pain through music within 15 min
	Voice and speech functions (articulation, fluency)	For child to engage in 30 min of rhythmic speech cueing
Activity and Participation/Activity Limitations and Participation Restrictions	General tasks and demands (daily routines, handling stress, managing behavior)	For child to engage in musical activities 3/5x without crying
	Communication (receptive, productive, using communication devices)	For child to clearly communicate yes with a head nod and no with the shaking of head 3/5x during session
	Interpersonal relationships (informal and formal relationships)	Tolerate 15 min of receptive music listening to increase tolerance of unfamiliar people
	Major life areas (education, work, engagement in play)	For child to engage in musical play through 20-min session
	Community, social, and civic life (community/social activities, recreation/leisure)	For child to engage in musical play for 30 min for normalization of environment

## Data Availability

Raw data supporting the conclusions of this article will be made available by the authors, without undue reservation, to any qualified researcher.
